# Corilagin enhances the anti-tumor activity of 5-FU by downregulating the expression of GRP 78

**DOI:** 10.1038/s41598-023-49604-1

**Published:** 2023-12-19

**Authors:** Simin Li, Xinquan Li, Xiliang Yang, Yumeng Lei, Mingxin He, Xiaochen Xiang, Qingming Wu, Hongyun Liu, Jiadun Wang, Qiang Wang

**Affiliations:** 1 Institute of Infection, Immunology and Tumor Microenvironment, Hubei Province Key Laboratory of Occupational Hazard Identification and Control, Medical College, Wuhan Asia General Hospital, Wuhan University of Science and Technology, Wuhan, 430065 China; 2 School of Basic Medicine, Hubei University of Science and Technology, Wuhan, 437100 China

**Keywords:** Cancer, Cell biology, Diseases

## Abstract

Colorectal cancer is one of the most common malignancies worldwide. Although initially effective, patients who receive chemotherapy ultimately experience various complications and develop chemo-resistance, leading to cancer recurrence. Therefore, we aimed to find a drug with good efficacy and low toxicity that could enhance the treatment with 5-Fluorouracil (a commonly used clinical drug) and reduce its dosing. Corilagin, an anti-tumor natural product, has received widespread attention. Glucose regulated protein 78 (GRP78) is overexpressed in colorectal cancer cells and plays a key role in the proliferation, migration and drug resistance of cancer cells. Importantly, GRP78 can affect the apoptosis induced by 5-fluorouracil in CRC cells. In the present study, we determined the synergistic anti-tumor activity of the combination treatment by cell proliferation assay, apoptosis assay, fluorescent staining, cell cycle analysis, WB and PCR assays. This synergistic effect was associated with S-phase blockade, intracellular reactive oxygen species production and downregulation of GRP78. Taken together, our results indicate that Corilagin acts as a potentiator of 5-fluorouracil and may have therapeutic potential for patients with CRC.

## Introduction

According to statistics, colorectal cancer (CRC) remains the third most prevalent malignancy and the second most deadly^1^. The incidence of early-onset CRC is also increasing year by year^2^. The large number of CRC cases poses a growing global public health challenge. At present, surgical resection, radiotherapy and chemotherapy have become primary means of treating CRC^3^. However, early diagnosis is still difficult because there are no obvious symptoms in the early stage of CRC. In patients with intermediate to advanced disease, surgical treatment is not effective and the disease tends to repeat^4^. Acquired drug resistance in the course of chemotherapy is also key to treatment failure^5^.

5-Fluorouracil (5-Fu), an antimetabolic drug, is a commonly used first-line chemotherapeutic drug in the clinical treatment of tumors^6^. It has a good therapeutic effect on a variety of cancers, including CRC, breast cancer, gastric cancer and liver cancer. However, most patients treated do not achieve complete eradication of tumor cells, leading to recurrence and poor outcomes after 5-FU treatment^7^. In recent years, the progressive increase in drug resistance and the corresponding dose-dependent toxicities has limited its clinical application^8^. Therefore, it is of great significance and practical value to find a drug with good efficacy and low toxicity or one that can improve the efficacy of chemotherapeutic drugs and reduce their use.

In recent years, natural products have gradually entered a new field of vision of tumor therapy. Corilagin is a reverse gallic acid tannin that can be extracted from a variety of Chinese herbs such as Longan, *Phyllanthus urinaria* L. and *Phyllanthus emblica* L.^9^ Most importantly, the long history of Traditional Chinese Medicine use has shown that Corilagin has low adverse or toxic, as well as mild drug activities. Many studies have revealed that Corilagin has good antiviral and antitumor activity with low toxicity to normal cells and tissues^10,11^. However, the anti-tumor activity mechanism of Coliragin was not fully understood and the research on CRC was lacking. Moreover, the hepatoprotective effect of natural drugs has also attracted a lot of attention in oncological chemotherapy and drug combination therapy. Previous studies have confirmed that Corilagin protects liver tissues and restores liver function, which can alleviate clinical symptoms and shorten the course of liver disease in patients with liver disease^12,13^. Therefore, the combination of natural products with 5-FU therapy may have synergistic advantages in reducing dose, improving efficacy, and reducing or delaying drug resistance as a novel strategy for oncology treatment.

GRP78 (glucose regulatory protein) was observed to be highly expressed in CRC samples after applying TCGA dataset analysis. GRP78, a critical cellular chaperone protein, plays an important role in maintaining the stability of the endoplasmic reticulum (ER), binding to the endoplasmic reticulum Ga^2+^, and assisting the proper folding and assembly of proteins. Recent studies have demonstrated that it also acts in tumor cell growth, infiltration, metastasis and apoptosis^14,15^. Silencing of GRP78 resulted in a suppression of CRC growth by the downregulation of vascular endothelial growth factor receptor-2/vascular endothelial growth factor (VEGFR2/VEGF) pathway^16^. Chang et al. also demonstrated that downregulation of GRP78 increased chemotherapeutic drug-induced apoptosis by inhibiting survival signaling through both the Akt pathway and the activation of PP2A^16^. GRP78 also participates in the resistance of tumor cells to unfavorable environments. GRP78 regulates intracellular reactive oxygen species (ROS) levels by modulating stress-related signaling pathways^17^. Besides, the possibility of CRC resistance to chemotherapy is substantially increased. Downregulation of GRP78 inhibited cell proliferation and increased the sensitivity of CRC to chemotherapeutic agents^18^. Therefore, therapeutic approaches targeting GRP78 may improve the efficacy of anti-cancer drugs, reduce toxic side effects, and facilitate the treatment and recovery of tumor patients.

The purpose of our study was to investigate the synergistic effects of Corilagin (a natural product) and 5-Fu (a clinically used drug) in the treatment of CRC and their potential mechanisms. The anti-tumor activity of single-agent Corilagin, 5-Fu and the combination therapy were assessed by cell proliferation assay, apoptosis experiments, fluorescent staining, cell cycle analysis, WB and PCR. The results showed that Corilagin has strong pro-apoptotic and inhibitory cell migration activities on CRC cells. Corilagin enhanced the sensitivity of CRC cells to 5-Fu. This synergistic effect may be related to the downregulation of GRP78 and intracellular ROS production. In addition, Corilagin and/or 5-FU treatment blocked CRC cells in the S phase.

## Results

### Corilagin inhibited CRC cell proliferation and enhanced the antiproliferation effect of 5-Fu

**Figure 1:**
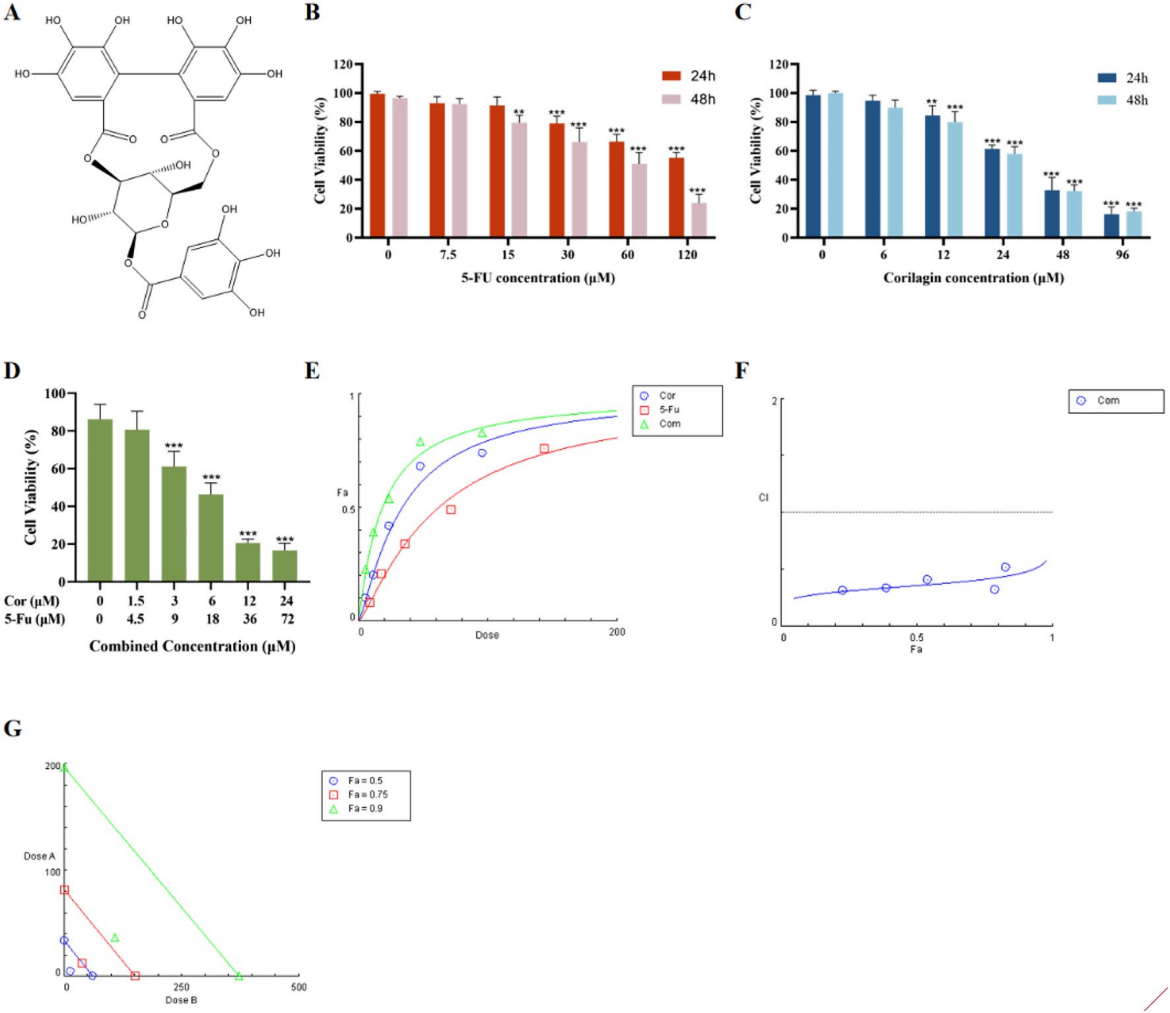
Corilagin and 5-FU synergistically inhibited the proliferation of HCT-8 cells. (**A**) The chemical structure of Corilagin was drawn using ChemDraw program. (**B**, **C**) HCT-8 cell line were treated with Corilagin (0, 6, 12, 24, 48, 96 μM) or 5-FU (0, 7.5, 15, 30, 60, 120 μM) in a dose range for 24 or 48 h. MTT assays were used to measure the cell viability. (**D**) HCT-8 cell line were treated with Corilagin and 5-FU (Corilagin + 5-FU 0, 1.5 + 4.5, 3 + 9, 6 + 18, 12 + 36, 24 + 72 μM) in a dose range for 48 h. MTT assays were used to measure the cell viability. (**E**) Dose Effect Curve of HCT-8 cells treated with Corilagin and/or 5-FU for 48 h was calculated by CompuSyn software using the Chou‐Talalay method. (**F**) Chou-Talalay method was used to determine the CI value, which shows the synergistic activity in the of drug combination. The CI for the combination of Corilagin and 5-FU was less than 0.5, denoting the significant synergistic effect. (**G**) The isobologram was used to evaluate the synergistic anti-value-added effect of coriligin after combination therapy with 5-FU. Synergism was indicated by the ED50 (Fa = 0.5) point falling below the summation line. The results are from 3 independent experiments and are presented as the means ± SD, each comprising five replicates per concentration level. Treated groups compared with the control group, **p < 0.01, ***p < 0.001.

Corilagin is an ellagitannin and a member of the tannin family. Corilagin was first isolated in 1951 from Dividivi extract and Caesalpinia coriaria, hence the name of the molecule^19^. In the following decade, a correct structural characterization was gradually obtained^20^. Structural identification was performed by comparing the 1H-NMR and 13C-NMR spectra (Supplementary Fig. S1A,B) with those reported in the literature^21,22^. The structures of the components identified are presented in Fig. 1A, and their spectral details are shown below. The compound was identified as corilagin.

Corilagin. ^1^H-NMR (400 MHz, DMSO-*d*_6_): *δ*_H_ 7.02 (2H, s, H-2′, H-6′), 6.56 (1H, s, H-6″), 6.50 (1H, s, H-6‴), 6.22 (1H, d, *J* = 7.20 Hz, H-1), 4.60 (1H, s, H-3), 4.34 (1H, t, *J* = 8.00 Hz, H-5), 4.25 (1H, s, H-4), 4.22 (1H, dd, *J* = 3.20, 11.21 Hz, H-6), 3.97 (1H, dd, *J* = 9.61, 10.01 Hz, H-6), and 3.88 (1H, d, *J* = 6.00 Hz, H-2); ^13^C-NMR (100 MHz, DMSO-*d*_6_): *δ*_C_ 167.6 and 167.2 (C=O), 165.3 (C=O), 146.1 (C-3′ and C-5′), 145.3 (C-3″), 145.2 (C-3‴), 144.8 (C-5″), 144.4 (C-5‴), 139.5 (C-4′), 136.0 (C-4″), 135.9 (C-4‴), 124.4 (C-2″), 123.5 (C-2‴), 119.2(C-1′), 116.3 (C-1″), 116.0 (C-1‴), 109.5 (C-2′ and C-6′), 107.4 (C-6″), 106.5 (C-6‴), glucose *δ*_C_ 92.6 (C-1), 78.1 (C-3), 76.8 (C-5), 72.1 (C-2), 64.4 (C-6), and 62.6 (C-4).

In the present study, HCT-8 cells were treated with Corilagin (0, 6, 12, 24, 48, 96 μM) and 5-FU (0, 7.5, 15, 30, 60, 120 μM) for a period of 24 h and 48 h of treatment, and the evaluation of cellular inhibition was performed through the MTT assay. As shown in Fig. 1B, treatment with various concentrations of 5-Fu significantly inhibited the proliferation of HCT-8 cells. ANOVA of repeated measures indicated that the efficacy of 5-Fu was dose- and time-dependent (*p *< 0.001). The IC50 value of 52.30 ± 6.80 μM for 48 h of treatment, whereas half inhibition was not achieved by treatment for 24 h over the same range of drug concentration gradients. The IC50 value after 24 h of treatment with a high concentration gradient of 5-FU (0, 30, 60, 120, 240, 480 μM) was 147.40 ± 4.58 μM (Supplementary Fig. S2). As shown in Fig. 1C, Corilagin inhibited the growth of HCT-8 cells in a dose-dependent manner (*p *< 0.001). The IC50 values were 31.69 ± 2.04 μM and 29.78 ± 1.25 μM for 24 h and 48 h of treatment, respectively.

Then, HCT-8 cells were treated with a fixed ratio concentration (1:3) of Corilagin and 5-FU in combination for 48h and cell survival was assayed (Fig. 1D). Subsequently, the combination index (CI) and drug effects (inhibition rate, Fa) were calculated by CompuSyn software using the Chou‐Talalay method^23^. The results showed that synergistic effects on HCT-8 cells exist with the combination of these two drugs (0.3 < CI < 0.7 for synergistic effect and CI < 0.3 for strong synergistic effect) (Fig. 1E–G). Corilagin in combination with 5-FU might reduce the concentrations of the two drugs, but retain the efficacy. The concentrations of Corilagin, 5-Fu, Corilagin in combination with -FU at the drug half inhibition rate (Fa = 0.5) were selected for follow-up experiments (Table 1).

**Table 1:** Dose–response correlation between 5-FU and Corilagin alone or in combination.

Compound	Dm (μM)	R	CI values			DRI			Combined dose (μM)
			Fa 0.25	Fa 0.5	Fa 0.75	Fa 0.25	Fa 0.5	Fa 0.75	Fa 0.5
Corilagin	33.67	0.99				8.53	7.32	6.29	4.60
5-FU	58.94	0.99				5.14	4.49	3.92	13.79
5-FU/Cor	18.38	0.99	0.31	0.36	0.41				

Subsequent treatment of SW480 cells with Corilagin (0, 6, 12, 24, 48, 96 μM) and 5-FU (0, 7.5, 15, 30, 60, 120 μM) for 48 h was used to validate the synergistic effect of the combination treatment. The IC50s for Corilagin and 5-FU were 31.66 ± 2.38 and 54.34 ± 4.29 μ M (Supplementary Fig. S3A,B). To investigate the antitumor effects of 5-FU plus Corilagin, SW480 cells were incubated with a fixed ratio concentration (1:3) of Corilagin and 5-FU in combination for 48h and cell survival was assayed. The results showed that the combination of 5-FU and Corilagin significantly inhibited the value-added of the SW480 cell line compared to the drug alone (Supplementary Fig. S3C). Subsequently, the CI and Fa were calculated by CompuSyn software using the Chou–Talalay method. The results showed that synergistic effects (CI < 0.7) on SW480 cells exist with the combination of these two drugs (Supplementary Fig. S3D–F). Noteworthy, drug combination treatments are well tolerated in normal cells. Compared to CRC cell lines (HCT-8 and SW480 cells), Coriligin was only mildly toxic to the normal NCM460 cell line at the same concentration, and drug combination therapy was even less toxic to the normal NCM460 cell line (Supplementary Fig. S3A–C).

### Corilagin inhibited CRC cell migration and enhanced the migration-inhibiting ability of 5-FU

**Figure 2:**
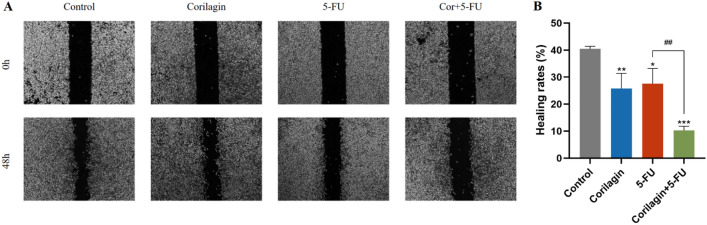
Corilagin and/or 5-FU inhibited CRC cell migration. (**A**) Cell migration ability was detected by wound repair assay. (**B**) Migration rate of HCT-8 cells under single or combined drug treatment. The results are from 3 independent experiments and are presented as the means ± SD. Treated groups compared with the untreated control, *p < 0.05, **p < 0.01, ***p < 0.001; Compared with the 5-FU group, ^##^*p* < 0.01.

Collective migration of cells is an indicator of tissue remodeling, including subcutaneous embryogenesis, wound healing, and cancer cell metastasis^24^. We next quantified cell migration by a wound healing assay. The wound area is more uniform and reproducible compared to the linear distance of the scratch. Therefore, the healing area was calculated by ImageJ software to evaluate the migration ability of HCT-8 cells in this experiment. Initial and final scratch areas were measured and the difference between the 0 and 48 h measurements was expressed as migration area. The results showed that the scratch healing rate in the Corilagin group (25.78 ± 4.57) % was significantly lower than that in the control group (40.53 ± 0.71) % (*P* < 0.001). The low-dose 5-FU and Corilagin synergistic group had a significantly lower scratch healing rate (10.26 ± 1.25) % than the control group (*P *< 0.001), and lower than the high-dose 5-FU group (27.59 ± 4.57) % (*P *< 0.001) (Fig. 2A,B).

### Corilagin and 5-FU synergistically promoted CRC cell apoptosis

**Figure 3:**
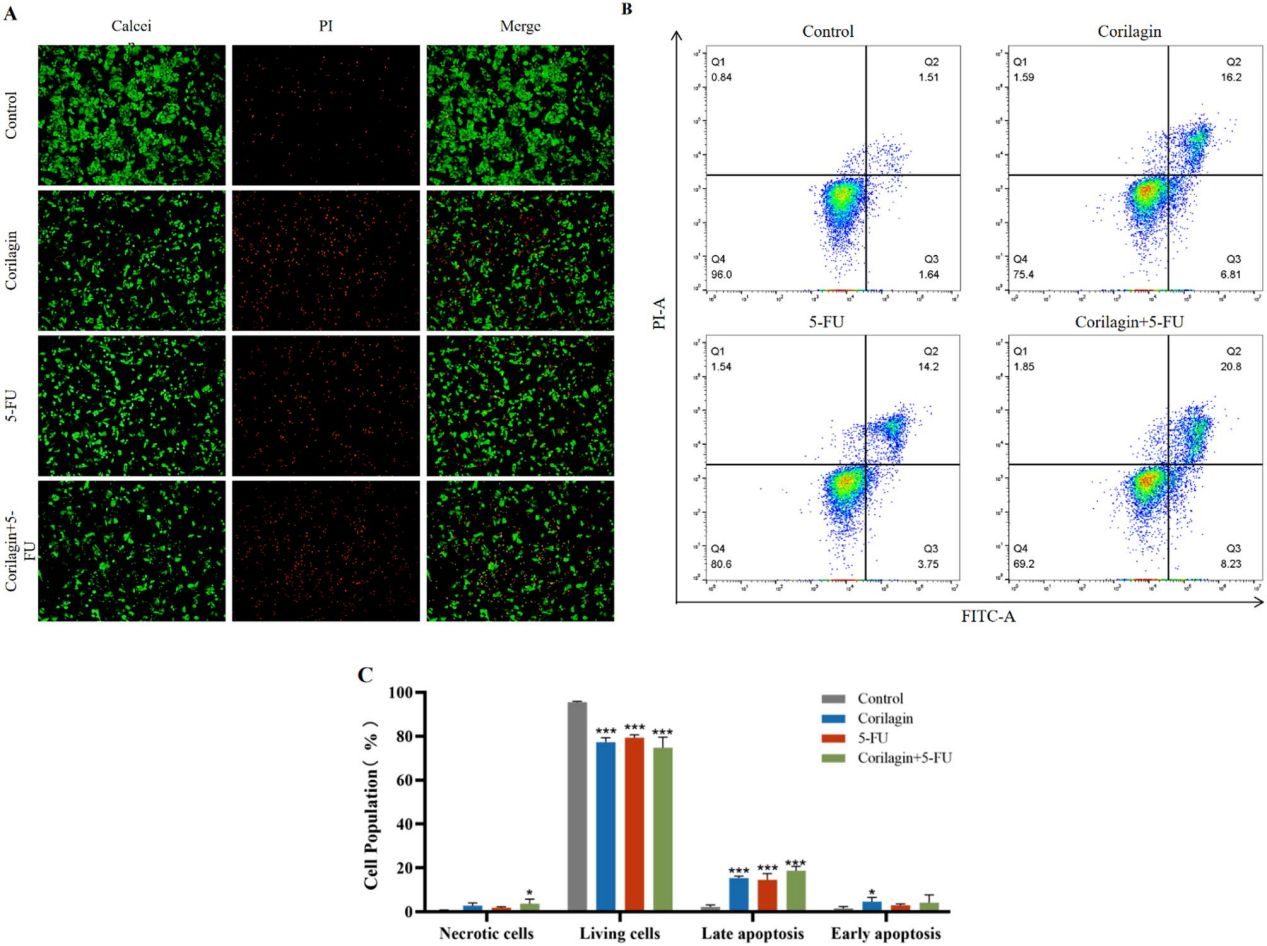
Corilagin and/or 5-FU induces apoptosis in HCT-8 cells. (**A**) The HCT-8 cells stained with Calcein AM/PI (Calcein AM labeled-green, PI labeled-red, respectively) were observed under fluorescence microscope to discriminate living and dead cells. (**B**) Apoptosis was assessed via Annexin V/PI dual-colour flow cytometry. Representative images of the cell distribution and percentage in the different quadrants indicating viable cells (Annexin V−/PI−) in the lower left, early apoptotic cells (Annexin V+/PI−) in the lower right and late apoptotic cells (Annexin V+/PI+) in the upper right. (**C**) Percentage of late apoptotic cells, early apoptotic cells, living cells and cell debris. The results are from 3 independent experiments and are presented as the means ± SD, each comprising three replicates per concentration level. Indicates a significant difference by Tukey’s test relative to their respective control (*p < 0.05, ***p < 0.001).

To study the effects of Corilagin and/or 5-FU on the activity and cytotoxicity of CRC cells, Calcein/PI staining, a DNA-specific fluorescent dye, was performed. HCT-8 cells were treated with drugs alone or in combination for 48 h and then stained. With fluorescence microscopy, HCT-8 cells in the treated group were observed to have morphological chromatin agglutination and bright blue apoptotic bodies similarly to the condensed and broken nuclei of apoptotic cells; and the fluorescence intensity of living cells decreased (Fig. 3A). The quantity of live cells in the Corilagin group was significantly lower than that in the control group. In addition, the decrease in the number of live cells in the combination treatment group showed a similar trend compared to the 5-FU group alone.

Subsequently, to determine the degree to which treatment of Corilagin and/or 5-FU resulted in apoptosis, we performed flow cytometry analysis with Annexin V-fluorescein isothiocyanate (FITC) and propidium iodide (PI) staining. Cells staining positive for Annexin V-FITC and negative for PI were considered to be undergoing apoptosis. The results illustrated that the apoptosis rate of the Corilagin group (18.84 ± 0.64) % was significantly higher than that of the control group (4.66 ± 0.89)% (*P *< 0.001). The apoptosis rate of the low-dose combination therapy group (19.61 ± 0.58) % was also significantly higher than that of the control group (*P* < 0.001), and had the same pro-apoptotic effect as the high-dose 5-FU group (16.94 ± 2.16)% (*P *> 0.05) (Fig. 3B,C).

### Corilagin coordinated with 5-FU arrests CRC cells in the S phase

**Figure 4:**
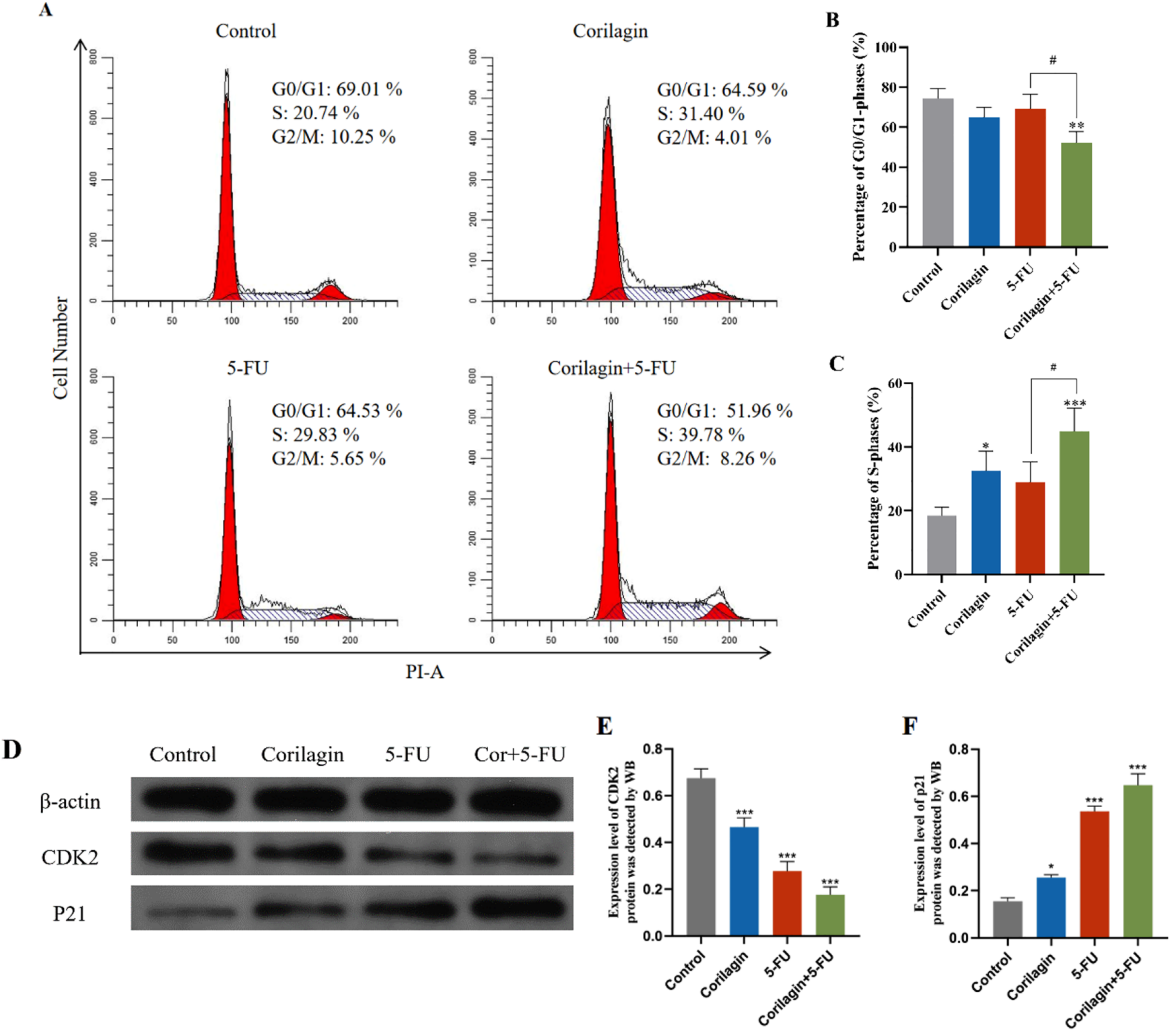
Corilagin and 5-FU synergistically induces S phase arrest in HCT-8 cells. (**A**) HCT-8 cells were treated with the indicated concentrations of Corilagin and/or 5-FU for 48 h. Cells were permeabilized by ethanol and stained with PI. Cell cycle progression was assessed by flow cytometry. Representative figures of the cell cycle distribution (G0–G1, S, and G2-M) show accumulation of Corilagin and/or 5-FU-treated cells in S phase. (**B**) The average percentage of the S phases is represented. (**C**) The average percentage of the G0/G1 phases is represented. (**D**) Representative images of WB reactions with protein extract from HCT-8 cells in coriligin and/or 5-FU treatment, showing the modulation of the expression of CDK2 and p21 involved in the G1-S transition of the cell cycle. Original blots are presented in Supplementary Fig. 4. (**E**) Expression level of CDK2 protein was detected by WB. (**F**) Expression level of p21 protein was detected by WB. The results are from 3 independent experiments and are presented as the means ± SD. Treated groups compared with the untreated control, *p < 0.05, ***p < 0.001.

To clarify whether the growth inhibitory effects induced by Corilagin and/or 5-FU are associated with cell cycle perturbation, we determined the cell cycle distribution of HCT-8 cells after 48 h of single and combination treatment. As the results showed, Corilagin alone led to an increase in the percentage of cells in the S phase compared to the control group (*P* < 0.05), with no significant difference in the percentage of cells in G1 and G2 phases. In the combined treatment group, the percentage of cells in the S phase was significantly increased compared to the control (*P* < 0.001) and the percentage of cells in G0/G1 phase decreased (*P* < 0.01). Compared with the 5-FU alone group, the low-dose combined treatment group showed a significant increase in the percentage of S phase cells (*P *< 0.05), suggesting that Corilagin promotes the blocking effect of 5-FU on the S phase of cells (Fig. 4A–C).

Beyond that, Western blot (WB) was implemented for measurement of the expressions of cyclin-dependent kinase 2 (CDK2) and CDK inhibitor (p21) that pertained to the cell cycle. Cyclin and CDKs are critical regulators of the cell cycle. The transition from G1 to S phase is significantly driven by Cyclin E and CDK2 and is regulated by P57, P21, and P27. CDK2 is activated by binding to Cyclin E, which drives the transition from late G1 phase to S phase^25^. P21, an important protein inhibitor in the cell cycle, can also bind to CDK, resulting in the blockage of cell cycle progression^26^. In this study, HCT-8 cells were treated with the indicated concentrations of Corilagin (33.6 μM), 5-FU (58.9 μM) and combination treatment (Corilagin+5-FU 4.6 + 13.8 μM) for 48 h, and then the WB assay was performed to evaluate protein expression levels. The results showed that the combination treatment resulted in a significant down-regulation of CDK2 protein levels and a significant up-regulation of p21 protein levels (Fig. 4D–F). 5-FU and Corlagin promote the cellular transition to S-phase by up-regulating p21 and reduce CDK2 to block the transition to the next step in S-phase, which is similar to that in Fig. 4A.

### Combination therapy of Corilagin and 5-FU promotes the accumulation of intracellular of ROS

**Figure 5:**
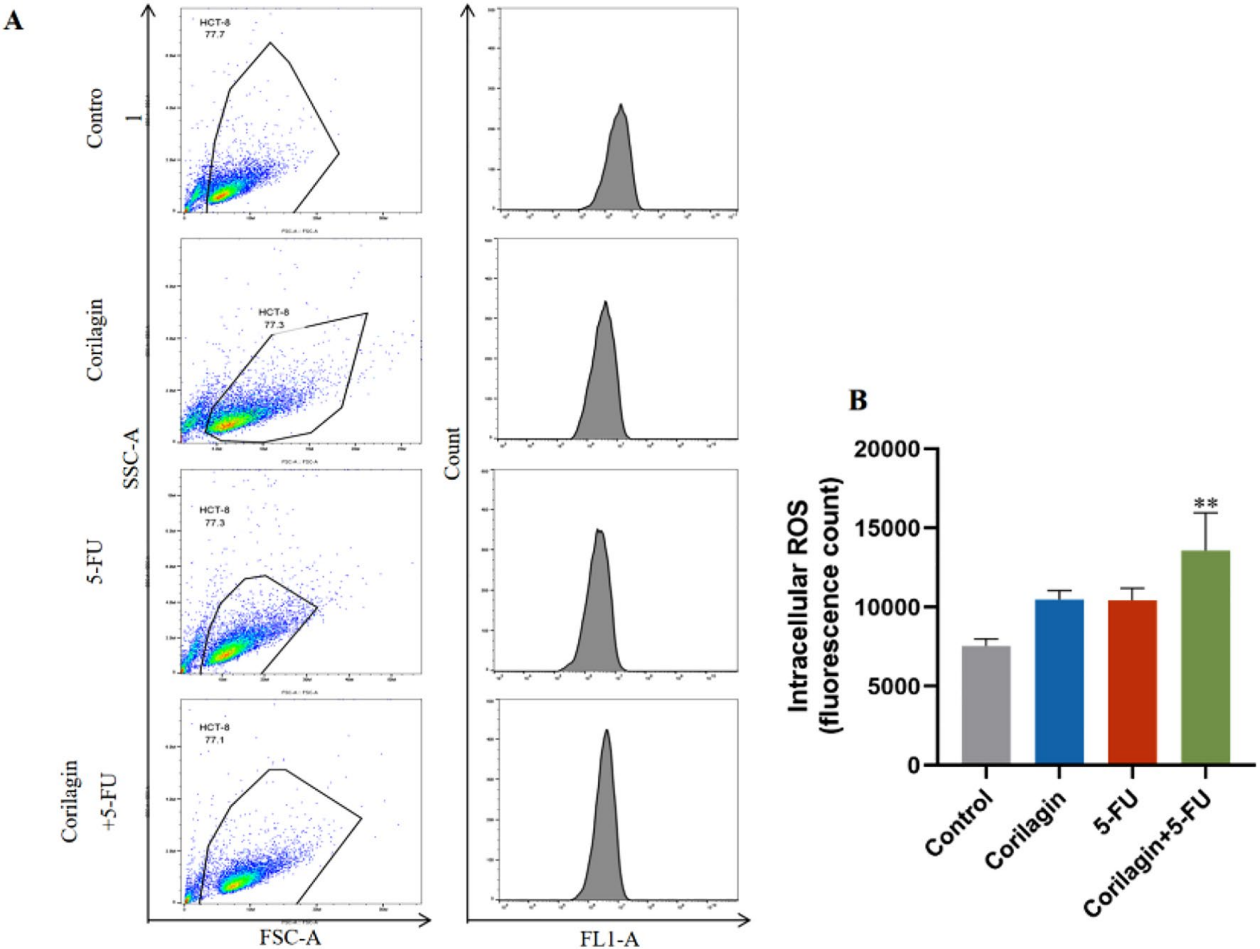
Combination treatment increased ROS production in HCT-8 cells. (**A**) The ROS level was detected by DCFH-DA probe assay. FlowJo software was used to fit the data. (**B**) ROS is displayed as a histogram in HCT-8 cell lines. The results are from 3 independent experiments and are presented as the means ± SD. Treated groups compared with the untreated control, **p < 0.01.

Increased oxidative stress is one of the causes of cell cycle arrest, and ROS play an important role in intracellular signal transduction and redox homeostasis^27^. The DCFH-DA probe labeled CRC cells treated with drugs detects the redox state of the cells by detecting changes in fluorescence. The results suggested that Corilagin promoted ROS production in HCT-8 cells, and the combination therapy of Corilagin and 5-FU significantly increased the production of ROS in HCT-8 cells (*P* < 0.01) (Fig. 5A,B).

### Combination therapy of Corilagin and 5-FU downregulates expression of GRP78

**Figure 6:**
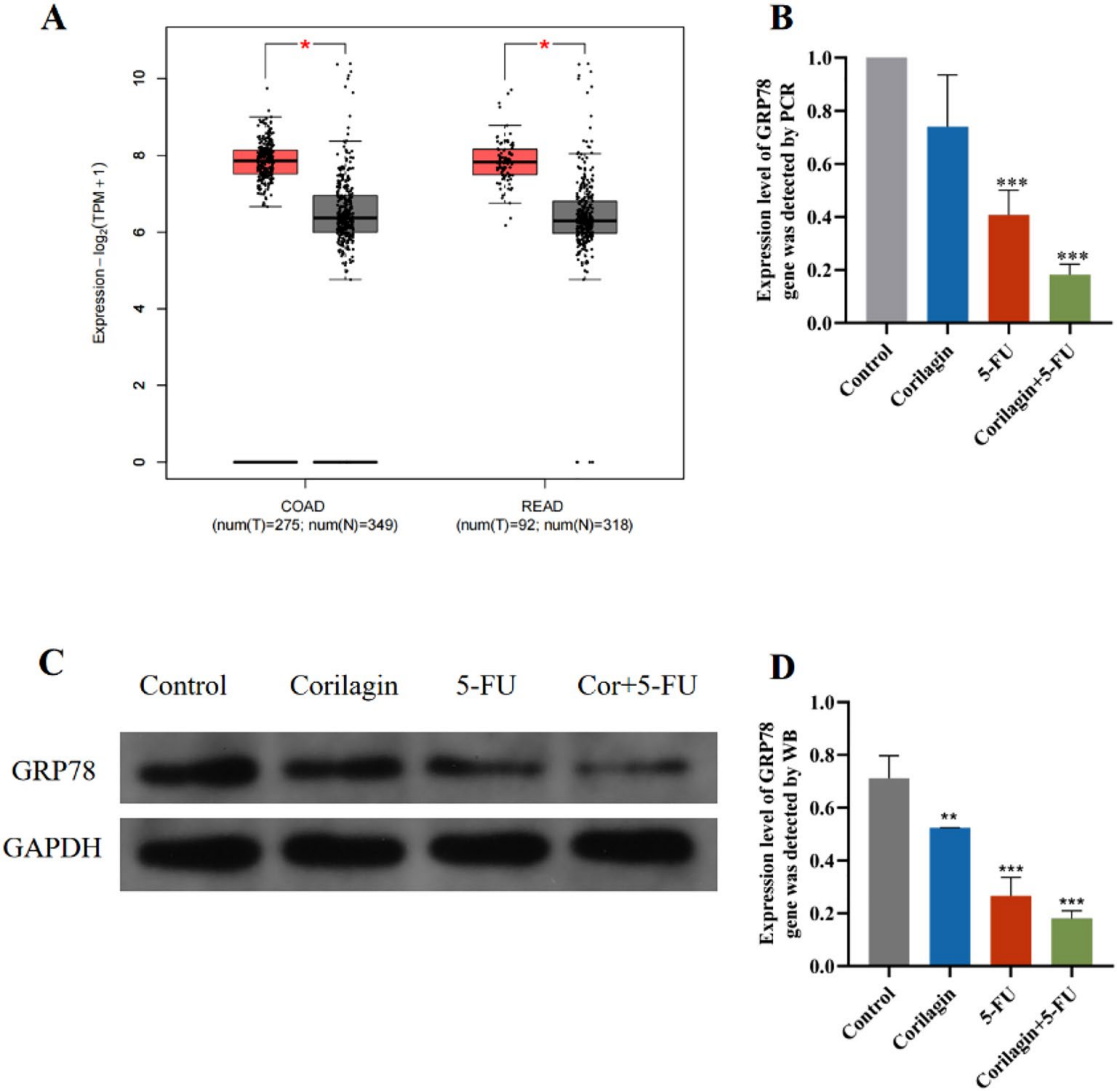
Combination treatment reduces GRP78 expression in HCT-8 cells. (**A**) The differential expression analysis of GRP78 mRNA in CRC (including COAD and READ) using GEPIA 2 (Expression DIY platform) based on TCGA and GTEx databases. Red represents tumor tissues and grey represents normal tissues. |Log2FC|> 1 and *P* value < 0.01 was set as the cutoff. (**B**) RT-PCR detection of GRP78 expression in the control group, the corilagin treatment group, the 5-FU treatment group, and the drug combination treatment group, and the bar graph was plotted using GraphPad Prism 8. (**C**, **D**) WB detection of GRP78 expression in the control group, the corilagin alone treatment group, the 5-FU alone treatment group and the drug combination treatment group, and bar graphs were plotted using GraphPad Prism 8. Original blots are presented in Supplementary Fig. 5. The results are from 3 independent experiments and are presented as the means ± SD. Treated groups compared with the untreated control, **p < 0.01, ***p < 0.001.

Currently, the molecular targets of Corilagin against CRC are still unknown. GRP 78 is mainly located in the endoplasmic reticulum as a molecular chaperone and is considered an important participant in cancer progression because of its role in promoting tumor cell proliferation, survival and tumor angiogenesis^28^. First, TCGA and GTEx database were utilized to explore the expression of GRP78 between tumor and normal tissues. GEPIA 2 (http://gepia2.cancer-pku.cn/#analysis) is a multi-dimensional cancer genomic dataset comprising a large amount of data from TCGA and GTEx databases^29^. The database contains 349 samples of normal tissues and 275 samples of tumor tissues with COAD, and 318 samples of normal tissues and 92 samples of tumor tissues with READ. The results showed (Fig. 6A) that the mRNA level of GRP78 in CRC (including COAD and READ) was higher than that in normal tissues. Red represents tumor tissues and gray represents normal tissues. Subsequently, the effect of Corilagin and/or 5-FU on GRP 78 levels in HCT-8 cells was observed by PCR and WB test. The results showed that the GRP 78 levels were significantly lower in the Corilagin group and 5-FU group than in the control group. GRP78 levels were also significantly lower in the combination treatment group than in the control group, and the reduction was more pronounced than in the single-agent group (Fig. 6B–D). According to our current results, there is a direct interaction between Corilagin and GRP 78. Corilagin enhanced the efficacy of 5-FU targeting human CRC by promoting the regulatory effects of 5-FU on GRP 78 protein expression.

## Discussion

The antimetabolite 5-FU is an important component of CRC chemotherapy. 5-FU-based therapy has significantly improved CRC outcomes and survival rates. However, these benefits are often compromised by chemoresistance and side effects from overdosing or severe life-threatening toxicity^30^. Therefore, there is an urgent need to develop effective combination therapies to enhance the antitumor activity of 5-FU in the clinical treatment of CRC. In our study, we demonstrated that the combination of low-toxicity natural compounds with 5-FU significantly and synergistically inhibited the growth of CRC in vitro.

As a natural product that can be extracted in large quantities, Corilagin has been proved to have a variety of pharmacological activities in the past two years, such as anti-inflammatory, anti-tumor and hepatoprotective properties^31,32^. Moreover, since natural substances tend to have low toxicity to normal tissues, natural plant extracts are natural targets for alternative therapies. Some proprietary Traditional Chinese medicines containing Corilagin have been used in clinical applications for the treatment of rheumatoid arthritis, viral hepatitis B, chronic liver disease and other diseases^33^. However, there are no previous toxicity studies related to HCT-8 cell viability, and the effects and mechanisms of pro-apoptosis in CRC cells have not been clarified. In our study, it was first shown that Corilagin significantly inhibited cell proliferation in CRC cell lines (HCT-8 and SW480) in a concentration-dependent manner. Furthermore, the results showed that CRC cell lines exhibited higher sensitivity to Corilagin treatment compared to the 5-FU treatment. As with the current research, it has been reported that Corilagin has good antitumor activity in different cancer cells^34^. Noteworthy, Coriligin treatments are well tolerated in normal cells. At the same concentration, Coriligin had a reduced inhibitory effect on the value-added of normal NCM 460 cells compared to the CRC cell lines. We have also demonstrated through migration assay that Corilagin has great potential to prevent tumor metastasis. It is also proved that Corilagin has good anti-tumor activity in the apoptosis, migration and invasion of CRC cell lines.

In general, chemotherapy resistance is the main cause of postoperative recurrence and metastasis in CRC. Several studies have suggested that multiple factors may contribute to 5-FU resistance, including drug target mutations, enhancement of drug inactivation and ER stress^35,36^. GRP78 has been widely studied as a biomarker of ER stress; its high expression has been associated with drug resistance in several tumor cells^37,38^. In our study, the combination treatment of corilagin and 5-FU significantly down-regulated GRP78 levels in CRC cells, which may prevent the development of drug-resistance. This result was also supported by a study that silencing GRP78 improved the sensitivity of CRC cells to 5-FU, resulting in apoptosis^18^. Moreover, long-term or large doses of 5-FU are prone to drug resistance and a variety of adverse reactions, which severely limits the survival time and quality of life of colorectal cancer patients. Therefore, this study focused on achieving the same antitumor effect while reducing the dose of Corilagin and 5-FU used, which provides new therapeutic ideas to avoid resistance caused by high-dose 5-FU treatment. This is consistent with the existing clinical treatment protocols, and the combination of commonly used clinical drugs with low-toxicity natural products has promising clinical prospects.

To elucidate the underlying mechanisms of the antiproliferative effects of Corilagin and/or 5-FU, three main hypotheses were considered: regulation of intracellular oxidative stress, cell cycle arrest and activation of apoptosis. In recent years, ROS-mediated apoptosis has been observed in various types of cancers^39^. Therapeutic strategies that enhance ROS production may deplete the defenses of the antioxidant system, thereby effectively killing cancer cells and overcoming drug resistance^40,41^. The antioxidant properties of Corilagin have been described, with ROS accumulation possibly promoting apoptosis in Corilagin exposed tumor cells^34,42^. In our study, we demonstrated that Corilagin induced a slight increase in ROS levels in HCT-8 cells, with no significant difference in the results. Therefore, we speculate that inhibition of cell growth may be mainly through some other modality, such as the promotion of apoptosis and inhibition of the cycle in HCT-8 cells. Interestingly, Corilagin and 5-FU had a significant ROS accumulation at low doses, which should partially explain the synergistic effect of the combination therapy anti-proliferative and suggests that Corilagin can promote 5-FU-driven ROS accumulation.

In addition to anti/pro-oxidant activity, cell cycle arrest is also a vital factor affecting cell growth. Previous studies have found that many natural compounds inhibit the viability of cancer cells by promoting cell cycle arrest. Our study demonstrated that Corilagin blocks cell cycle progression in S phase, which was consistent with the results of the MTT assay. Therefore, we suggest that Corilagin inhibits the value-added of the HCT-8 cell line by inducing cell cycle arrest. Other studies have found that Corilagin inhibits U251 cells in the mitotic G2/M phase, while U251 stem cell-like cells are blocked in S phase^43^. Gu et al. also reported that Corilagin can inhibit CCA cell proliferation by inducing G2/M phase arrest, but no significant change in the MZ-Cha-2 cell cycle^44^. This difference in cell cycle inhibition may be related to differences in tumor cell lines, but the exact mechanism remains to be elucidated.

Jeverson and coworkers found that Corilagin decreased p-cdc2 (Tyr15) and cyclin B1 levels in ovarian cancer cells (Hey and SKOv3ip), which may be a molecular mechanism associated with cell cycle arrest^45^. Corilagin can also downregulate the expression of p-Akt, which enhances the ubiquitination and degradation of p53 and has a critical role in cell cycle inhibition^46^. In the present study, drug treatment in HCT-8 cells resulted in down-regulation of CDK2 protein levels and up-regulation of the levels of the cell cycle regulator p21. The increased levels of p21 protein expression may inhibit the formation of the cyclin A-CDK2 complex and contribute to cell cycle arrest in S phase. S-phase progression is controlled by the binding of CDK2 to cyclin A or cyclin E, which is further activated by Cdc25A mediated dephosphorylation, therefore any perturbations that affect the complexes formation or activation would result in S-phase cell cycle arrest^47^. Moreover, P21, a key cell cycle regulatory protein that governs cell cycle progression from G1 to S phase, can regulate cell proliferation, growth arrest and apoptosis^48^. The cell cycle protein-dependent kinase inhibitor p21 was previously shown to inhibit the activity of the cell cycle proteins CDK2, CDK1 and CDK4/6 complex, contributing to cell cycle arrest in S phase^25,49^.

In addition, we also found that the combination treatment stimulated a more significant increase in the S-phase cell population in the HCT-8 cell line compared to the 5-FU treatment alone. 5-FU is an atypical cell cycle-specific drug, which, in addition to acting mainly in the S phase, also has effects on cells in other phases. On the one hand, it blocks DNA replication by inhibiting thymidine synthesis, leading to cell cycle arrest in S phase^50^. A study demonstrated that 5-Fu, an anticancer drug that primarily targets S phase, caused cell cycle arrest in S phase in HCT116 cells^51^. 5-Fu can block the cell cycle in S phase by inhibiting thymidylate synthase, and its metabolites can also be doped into the DNA and RNA of the cells cycling through the cell^52^. On the other hand, it also induces nucleolar stress by doping ribosomal RNA and interfering with subsequent ribosomal RNA processing. This leads to p53 activation, which induces G1 arrest and apoptosis^51^. The prolonged G1 and S phases may provide more time for 5-Fu to induce DNA damage in cancer cells. Therefore, we speculate that Corilagin partially enhances the chemotherapeutic effect of 5-FU on CRC HCT-8 cells via cell cycle blockade.

Moreover, several studies have revealed that Corilagin and 5-FU can activate apoptosis in cancer cells^53,54^. Apoptosis is a gene-coded “suicide” procedure that plays a pivotal role in diverse physiological and pathological processes^55^. The results of flow cytometry showed that Corilagin and/or 5-FU treatment of HCT-8 cells increased the percentage of both early and late apoptotic cells, which may be another mechanism for its tumor suppressive ability. Apoptosis is mainly regulated by death receptor-mediated extrinsic, mitochondria-mediated intrinsic and endoplasmic reticulum stress-induced apoptotic pathways^55^. Corilagin increased the protein levels of cleaved cystatin-3 and Bak and decreased the levels of Bcl-xl, which sustained the stimulation of the intrinsic apoptotic pathway^46^. Research by Xu and his coworkers also demonstrated that Corilagin induces cysteine-dependent apoptotic cell death in human gastric cancer^34^.

The endoplasmic reticulum stress-induced apoptotic pathway is accompanied by the activation of GRP78, CHOP, p-IRE1, and TRAF2, leading to cell death. Several studies have shown that GRP78 is one of the most important and representative stress proteins in endoplasmic reticulum stress (ERS)^31^. It is abundantly induced and expressed in tumor tissues and participates in tumor cell proliferation, anti-apoptosis, metastasis, drug resistance and other biological activities. Therefore, GRP78 is considered as a potential drug target for cancer intervention. The in vitro experiments confirmed that knockdown of GRP78 could inhibit tumor formation and progression^56^. In our study, we demonstrated that both mRNA and protein expression of GRP78 were downregulated after Corilagin treatment of HCT-8 cells. We speculated that Corilagin may be involved in antitumor effects by modulating endoplasmic reticulum stress through reducing the accumulation of intracellular GRP78. Additionally, we observed that the combination treatment stimulated a more significant reduction in GRP78 expression in HCT-8 cell lines compared to the drugs alone. As endoplasmic reticulum stress induces 5-FU resistance in human colon cancer cells^57^, downregulation of GRP78 levels by combination therapy helped to alleviate the adverse outcome of 5-FU resistance and enhanced the modulatory effect of 5-FU on ERS. The transition from ERS to apoptotic response is unclear, but it does seem to be dependent on ROS^16^. Furthermore, the inhibition of proliferation in CRC cells by GRP78 knockdown was associated with S-phase blockade, reduced G1/S transition, and downregulation of AKT and ERK1/2 (key cell cycle regulatory proteins) phosphorylation^18^. This also agrees with our results. Notably, previous studies reported that the specific inhibition of GRP78 expression suppressed G1/S transition-related cyclins (D1, E1, and E2) and cyclin-dependent kinase (CDK4 and CDK6) protein expression^58^. Therefore, the molecular mechanism by which GRP78 inhibitors induce CRC cell cycle arrest and inhibit proliferation requires our further investigation.

In the present study, the antitumor effects of Corilagin were associated with cell cycle blockade, intracellular ROS generation and down-regulation of GRP78. We found that bioactive substance Corilagin has a good synergistic effect on CRC, and its combination with clinical chemotherapeutic drug 5-FU may be a new strategy to enhance anti-tumor efficacy and minimize drug side effects.

## Materials and methods

### Preparation of Corilagin

Take 2.0kg of dried ripe fruit of Phyllanthus emblica L, crushed, extracted by maceration with 80% ethanol at room temperature, and concentrated under reduced pressure to obtain 200.6g of ethanol crude extract. The extract was extracted with n-butanol and eluted by ODS column chromatography with a methanol-water gradient (20%–40%–60%–80%–95%, v/v). The same fractions were combined according to the results of thin-layer chromatography and separated on a Sephadex LH-20 column (MeOH elution), eluted by semi-preparative HPLC (mobile phase MeOH /H2O containing 0.1% formic acid, 5–100% MeOH) to give the monomeric compound (1.2 g). The compound was identified by 13C-NMR and 1H-NMR.

### Cell culture

The human CRC cell lines (HCT-8 cells and SW480 cells) were purchased from the Wuhan Cell Collection, Wuhan, China. HCT-8 cells are the human colon cancer cell line that is keratin positive; expresses CEA and alkaline phosphatase; and is tumorigenic in nude mice. The growth mode is adherent. SW480 cells are human colorectal adenocarcinoma cells that are polygonal and adhere to the stroma in scattered patchy growth. The cells were cultured in RPMI 1640 (GIBCO, Invitrogen, USA) in a constant temperature incubator of 37 °C and 5% CO2. The RPMI 1640 was supplemented with 10% fetal bovine serum, 1% penicillin and streptomycin double antibodies solution (GIBCO, Invitrogen, USA).

### Reagent preparation of 5-FU

5-FU (F6627) was purchased from Sigma-Aldrich (MO, USA). 1 mg of 5-Fu powder was weighed on an electronic balance and dissolved in 32 µL Dimethyl sulfoxide (DMSO) (Solarbio, China) as storage solution. The concentration of this storage solution is 240 mM. Stored in − 20 °C refrigerator. For drug intervention, RPMI 1640 was used to prepare a liquid with less than 0.1% DMSO.

### Cell viability assay

The MTT (methyl thiazolyl tetrazolium) (Zhongshan Lianjiu Biotechnology Co, China) assay is a method for detecting cell survival and growth. The succinate dehydrogenase in the mitochondria of living cells can reduce the exogenous MTT to water-insoluble blue-violet Formazan and deposit it in the cells, while the dead cells do not have such a function. Within a certain cell number range, the amount of MTT crystal formation is proportional to the number of cells. The light absorption value is measured by an enzyme immunoassay instrument at the wavelength of 490nm, which can indirectly reflect the number of living cells. HCT-8 cells were seeded in 96-well plates at 5 × 10^3^ cells in 100 μl per well, incubated at 37 °C, saturated humidity and 5%CO2. Gradient concentrations of corilagin and/or 5-Fu were then added to the appropriate wells, and an equal volume of RPMI 1640 medium was added to the control group. After 24 and 48 h of drug treatment, the old medium was removed and MTT dilution was added. 4 h later, MTT was replaced with 150 μL of DMSO. The absorbance (OD) at 570 nm was measured using an enzyme marker. Cell viability [%] = (total number of cells - number of dead cells)/(total number of cells) × 100. IC50 values were calculated by GraphPad Prism 8 software (GraphPad Software, San Diego, CA) from MTT assay data. Results are from 3 independent experiments with 5 replicates per concentration level.

### Quantitative assessment of cell viability based on microscopy and flow cytometry

#### Microscopic measurement (Calcein-AM/PI double staining)

HCT-8 cells were seeded in 96-well plates at 5 × 10^3^ cells in 100 μl per well, incubated at 37 °C, saturated humidity and 5%CO2. Gradient concentrations of corilagin and/or 5-Fu were then added to the appropriate wells, and the corresponding volume of RPMI medium 1640 was added to the control wells. After 48 h of drug treatment, HCT-8 cells were collected. Dissolve Calcein-AM and PI solution (Beyotime, Shanghai) 1:1 in Binding buffer, add the configured mixed staining solution to the cell suspension, and incubate for 15 min at 37 °C. Calcein AM and propidium iodide (PI) are two fluorescent dyes that are used to detect live and dead cells, respectively. Then, the samples were washed three times in phosphate-buffered saline (1 × PBS). Live cells with yellow-green fluorescence and dead cells with red fluorescence were observed and imaged using an inverted fluorescence microscope (Nikon ECLIPSE TI, Nikon, Japan) at 490 nm and 545 nm excitation wavelengths. At least five visual fields were analyzed under fluorescence microscope for each sample.

#### Flow cytometric measurement (AnnexinV/PI double staining)

HCT-8 cells were seeded in 6-well plates at 2 × 10^5^ cells in 2000 μl per well, incubated at 37 °C, saturated humidity and 5%CO2. Gradient concentrations of corilagin and/or 5-Fu were then added to the appropriate wells, and RPMI medium 1640 was used instead of the control. After 48 h of drug treatment, collected HCT-8 cells. 5 μL of an 8 mM PI stock solution and 5 μL of a 4 mM AnnexinV-FITC (Zomanbio, Beijing) stock solution were added to 10 mL of sterile, tissue culture-grade PBS, and the solution was vortexed to ensure thorough mixing. The resulting working solution was used to stain the cells by incubation for 30 min at room temperature. Then, the samples were washed three times in phosphate-buffered saline (1 × PBS). Detected by BD FACSCelesta flow cytometry (Becton, Dickinson and Company, USA). The results were analyzed using FlowJo V10 software. Early apoptotic cells fluoresce green when stained with Annexin V-FITC and, on the fluorescence-activated cell sorting histogram, they show up in the lower right quadrant. Late apoptotic cells, when stained with both Annexin V-FITC and PI, give red-green fluorescence and are present in the upper right quadrant of the histogram. Necrotic dead cells, when stained with PI only, are present in the upper left quadrant. Results are from 3 independent experiments with 3 replicates per concentration level.

### ROS detection

HCT-8 cells were seeded in 6-well plates at 2 × 10^5^ cells in 2000 μl per well, incubated at 37 °C, saturated humidity and 5%CO2. Gradient concentrations of corilagin and/or 5-Fu were then added to the appropriate wells, and RPMI medium 1640 was used instead of the control. After 48 h of drug treatment, collected HCT-8 cells. HCT-8 cells were collected from each group after being treated with drugs. Add 300 ml of diluted DCFH-DA (Beyotime Biotechnology, Shanghai, China) to each well. Samples were incubated at 37 °C for 20 min and washed three times with PBS solution to remove DCFH-DA that did not enter the cells. DCFH-DA itself does not have fluorescence and can freely pass through the cell membrane. After entering the cell, it can be hydrolyzed by esterases inside the cell to generate DCFH. However, DCFH cannot penetrate the cell membrane, making it easy for the probe to be loaded into the cell. Reactive oxygen species within cells can oxidize DCFH to produce fluorescent DCF. By detecting the fluorescence of DCF, the level of intracellular ROS can be determined. Samples were analyzed by BD FACSCelesta flow cytometry (Becton, Dickinson and Company, USA), and the data were analyzed by FlowJo V10 software. Results are from 3 independent experiments with 3 replicates per concentration level.

### Cell cycle analysis

HCT-8 cells were seeded in 6-well plates at 2 × 10^5^ cells in 2000 μl per well, incubated at 37 °C, saturated humidity and 5%CO2. Then, the cells were treated with drugs for 48 h. The original medium was discarded, and the cells were harvested and fixed by 70% ice-cold (v/v) ethanol overnight at 4 °C. After that, the fixed cells were centrifuged and washed with cold phosphate-buffered saline (PBS), and then, they were further resuspended in a solution of 0.5 mL PI (Zomanbio, Beijing) for 30 min at 37 °C in the dark. The cells were determined by BD FACSCelesta Flow Cytometer (Becton, Dickinson and Company, USA), and the data were analyzed by modfit 3.1 software. Results are from 3 independent experiments.

### Wound healing

HCT-8 cells in the logarithmic growth phase were inoculated into a 6-well plate, and incubated at 37 °C until 90% of the cells covered the bottom of the well. A sample adding suction head (sterilized) was used to scratch perpendicular to the bottom of the plate. After drug treatment, observe the healing status of scratches under an inverted microscope (Olympus, Japan). The healing area was calculated by ImageJ software (National Institutes of Health (NIH), Bethesda, MD, USA) (https://imagej.net/ij/index.html). Cell migration rate = (initial scratch width - scratch width after 48 h)/initial scratch width × 100%. Results are from 3 independent experiments with 3 replicates per concentration level.

### Western blot analysis

HCT-8 cells were seeded in 6-well plates at 2 × 10^5^ cells in 2000 μl per well, incubated at 37 °C, saturated humidity and 5%CO2. Then, the cells were treated with drugs for 48 h. The original medium was discarded, and the samples were collected by lysing cells in RIPA lysis buffer (G2002, Servicebio, Wuhan). Part of the supernatant was taken and the protein concentration was measured by BCA method; 4X loading buffer was added into the rest of the supernatant for Western blot. The supernatant was incubated in boiling water for 5 min. The mass of protein loaded per lane was 40 µg. Each sample was size fractionated using SDS‐polyacrylamide gel electrophoresis (PAGE) and electro transferred onto PVDF membranes. After bolted with milk, the membranes incubated with primary antibodies (P21, 21KD, Affinity Bioscience, USA; CDK2, 34KD, Novus Biologicals, USA; β-actin, 42KD, Affinity Bioscience, USA; GAPDH, 37KD, Goodhere Biotechnology, Hangzhou; GRP78, 70-78KD, Proteintech, Wuhan) overnight at 4 °C and then blotted with horseradish peroxidase conjugated secondary antibodies (Horseradish peroxidase (HRP)) (Beyotime Biotechnology, China). The immunoblots were visualized using the ChemiDocTM XRS^+^ imaging system (Bio rad Company, USA). Results are from 3 independent experiments.

### RT-PCR analysis

HCT-8 cells were seeded in 6-well plates at 2 × 10^5^ cells in 2000 μl per well, incubated at 37 °C, saturated humidity and 5%CO2. Gradient concentrations of corilagin and/or 5-Fu were then added to the appropriate wells, and RPMI medium 1640 was used instead of the control. After 48 h of drug treatment, the original medium was discarded, and RNA was extracted from the transfected cells in the 6-well plate according to the Trizol method^59^ (Ambion Company, USA), and the total cell RNA was reverse transcribed into cDNA according to the kit instructions (Ambion, Beijing). 2 μl of the resulting cDNA pool was then used for amplification of the desired target using specific primers. Using cDNA as a template and GAPDH as an internal reference gene, qPCR amplification was performed according to the gene amplification instructions to detect the expression of the GRP78 gene (Tsingke Biotechnology, Beijing). The primer sequences, template cDNA, TB Green Mix, and RNase-free water in Table 2 were mixed to form an amplification system. The qPCR program consisted of 45 cycles of 10 s at 95 °C and 30 s at 60 °C, with a melting curve from 65 to 95 °C. Relative expression was calculated with the 2^−ΔΔCt^ method which refers to the ratio of the target genes to the housekeeping gene^60^. Results are from 3 independent experiments.

**Table 2:** Sequences of the primers used in the real-time PCR.

Gene	Primer	Sequence (5′-3′)	PCR products
Homo GAPDH	Forward	TCAAGAAGGTGGTGAAGCAGG	115 bp
	Reverse	TCAAAGGTGGAGGAGTGGGT	
Homo GRP78	Forward	AAGCAACCAAAGACGCTGGAACTAT	113 bp
	Reverse	GTTCTTCTCCCCCTCCCTCTTATCC	

### Statistical analysis

The data detected by flow cytometry were analyzed by FlowJo software and ModFit LT 5.0, and the scratch results were calculated by ImageJ software. Statistical analyses were performed by GraphPad Prism 8.0 software, one-way analysis of variance (ANOVA) and non-parametric tests were used to test for statistical comparison between groups. *P* <  0.05 was considered to be statistically significant (*p < 0.05, **p < 0.01, ***p < 0.001). The data (Error bars) are presented as the mean ± SD. Statistical differences between two or more groups were estimated with a two-tailed Kruskal-Wallis test or by ANOVA if normality was assumed based on the Shapiro-Wilk test. The combination index was calculated by CompuSyn software (https://www.combosyn.com/) using the Chou‐Talalay method^23^. All experiments were performed at least three times.

### Ethical statement

The plant material Phyllanthus emblica L. used in the experiment is a widespread species, which is not an endangered species, and its collection is in accordance with national regulations.

### Supplementary Information

Supplementary Figures.

## Data Availability

The data used in the current study are available upon request from authors. The corresponding author Qiang Wang could be contacted if anyone wants to request the data from this study.
