# Supplementary material for: Corilagin enhances the anti-tumor activity of 5-FU by downregulating the expression of GRP 78
Source: Sci Rep. 2023 Dec 19;13:22661. doi: 10.1038/s41598-023-49604-1 (PMC10730900; doi:10.1038/s41598-023-49604-1)
Supplement: Supplementary file 1 — Supplementary Figures. [file 41598_2023_49604_MOESM1_ESM.docx]

**Supplementary Information**

*for*

**Corilagin enhances the anti-tumor activity of 5-FU by inducing ERS and downregulating the expression of GRP 78**

Simin Li^1^, Xinquan Li^1^, Xiliang Yang^1^, Yumeng Lei^1^, Mingxin He^1^, Xiaochen Xiang^1^, Qingming Wu^1^, Hongyun Liu^2*^, Jiadun Wang^1*^, Qiang Wang^1*^

1 Institute of Infection, Immunology and Tumor Microenvironment, Hubei Province Key Laboratory of Occupational Hazard Identification and Control, Medical College, Wuhan Asia General Hospital, Wuhan University of Science and Technology, Wuhan, 430065, China

2 School of basic medicine, Hubei University of science and technology, Xianling 437100, China

*[Corresponding](mailto:corresponding.author@email.example): Qiang Wang (email: [wangqiang@wust.edu.cn](mailto:wangqiang@wust.edu.cn)); Hongyun Liu (email: [115550490@qq.com); Jiadun](mailto:115550490@qq.com；Jiadun) Wang (email: 392176771@qq.com).

**This supplementary file contains Supplementary Figure 1 to 5.**

**Supplementary Fig. 1. Structural Identification of Corilagin.**

**(A) The ^1^H NMR spectrum of Corilagin.** Frequency(MHz): 400.2100; Nucleus: 1H; Solvent: DMSO-d6. **(B) The ^13^C NMR spectrum of Corilagin.** Frequency(MHz): 100.6329; Nucleus: 13C; Solvent: DMSO-d6. The structures of the compounds were analyzed by 1H-NMR and 13C-NMR spectra.

Corilagin. ^1^H-NMR (400 MHz, DMSO-*d*_6_): *δ*_H_ 7.02 (2H, s, H-2', H-6'), 6.56 (1H, s, H-6"), 6.50 (1H, s, H-6'''), 6.22 (1H, d, *J* = 7.20 Hz, H-1), 4.60 (1H, s, H-3), 4.34 (1H, t, *J* = 8.00 Hz, H-5), 4.25 (1H, s, H-4), 4.22 (1H, dd, *J* = 3.20, 11.21 Hz, H-6), 3.97 (1H, dd, *J* = 9.61, 10.01 Hz, H-6), and 3.88 (1H, d, *J* = 6.00 Hz, H-2); ^13^C-NMR (100 MHz, DMSO-*d*_6_): *δ*_C_ 167.6 and 167.2 (C=O), 165.3 (C=O), 146.1 (C-3' and C-5'), 145.3 (C-3'') , 145.2 (C-3'"), 144.8 (C-5"), 144.4 (C-5'''), 139.5 (C-4'), 136.0 (C-4''), 135.9 (C-4'''), 124.4 (C-2"), 123.5 (C-2'''), 119.2(C-1'), 116.3 (C-1''), 116.0 (C-1'''), 109.5 (C-2' and C-6'), 107.4 (C-6''), 106.5 (C-6"'), glucose *δ*_C_ 92.6 (C-1), 78.1 (C-3), 76.8 (C-5), 72.1 (C-2), 64.4 (C-6), and 62.6 (C-4).


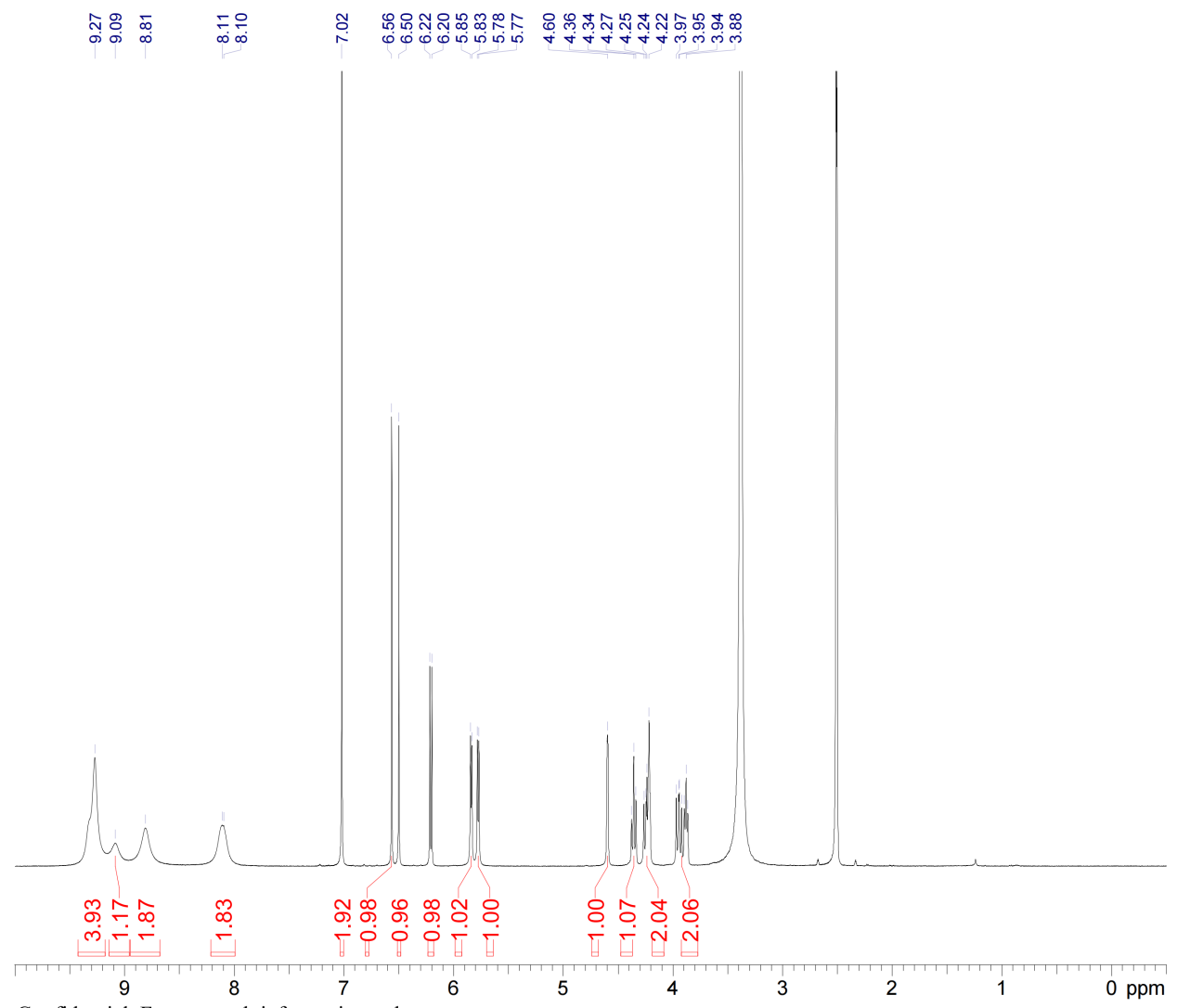


A


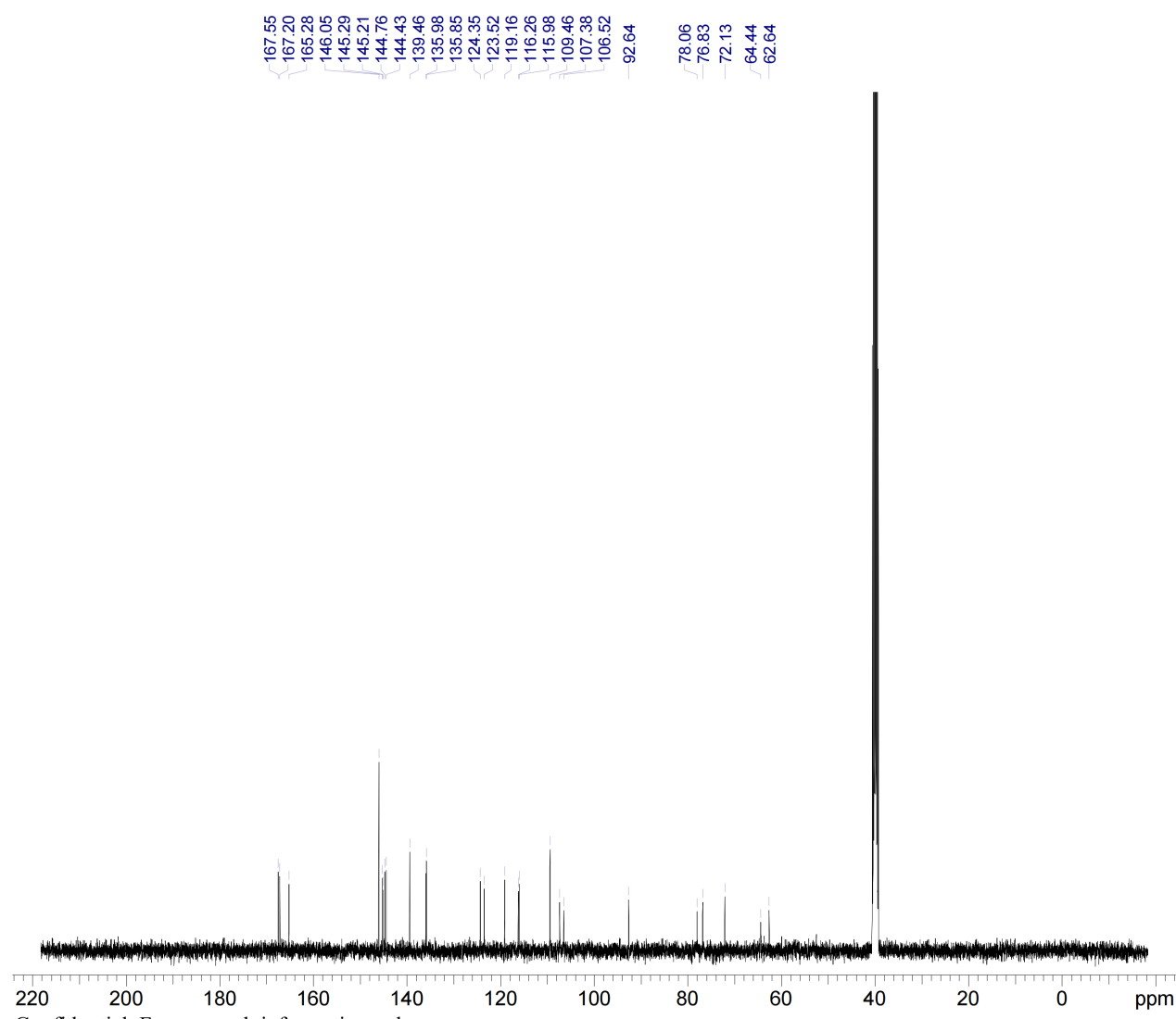


B

**Supplementary Fig. 2. The antiproliferation effect of 5-Fu.**

**(A) Cell viability of HCT-8 cells treated with specified concentrations of 5-FU for 24 hours.** The concentration gradient is 0, 30, 60, 120, 240, 480 μM. The IC50 value was 147.40 ± 4.58 μM. The results are from 3 independent experiments and are presented as the means ± SD, each comprising five replicates per concentration level. Treated groups compared with the control group, **p < 0.01, ***p < 0.001.

**A**

#
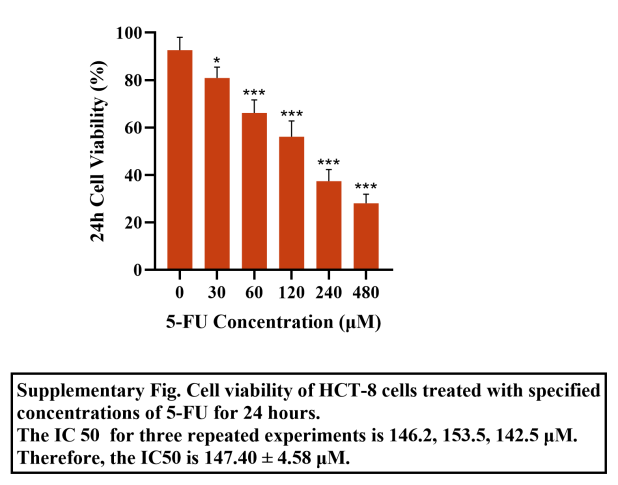


**Supplementary Fig. 3. The anti-proliferative effects of Corligin, 5-FU and combination treatments.**

**(A) Corligin on colorectal cancer cells (HCT-8 and SW480) and normal colorectal epithelial cells NCM460 cells.** HCT-8, SW480 and NCM460 cell lines were treated with the indicated concentrations of Corilagin (0, 6, 12, 24, 48, 96 μM) for 48 h, and then the MTT assay was performed to evaluate cell proliferation inhibition rates. The results showed that significant positive correlation between the concentration of Corligin and cell inhibition rate was observed in CRC cells (p<0.001). However, the toxic effect of Corligin on NCM460 cell lines was reduced compared to CRC cells. **(B) 5-FU on colorectal cancer cells (HCT-8 and SW480) and normal colorectal epithelial cells NCM460 cells.** HCT-8, SW480 and NCM460 cell lines were treated with the indicated concentrations of 5-FU (0, 7.5, 15, 30, 60, 120 μM) for 48 h, and then the MTT assay was performed to evaluate cell proliferation inhibition rates. The results showed that significant positive correlation between the concentration of 5-FU and cell inhibition rate was observed in CRC cells (p<0.001). In NCM460 cells, low concentrations of 5-FU (concentration <15 μM) did not significantly inhibit cell proliferation (p > 0.05), while high concentrations of 5-FU (concentration >30 μM) inhibited cell proliferation (p < 0.01). **(C) Combination treatment on colorectal cancer cells (HCT-8 and SW480) and normal colorectal epithelial cells NCM460 cells.** HCT-8, SW480 and NCM460 cell lines were treated with the indicated concentrations of combination treatment for 48 h, and then the MTT assay was performed to evaluate cell proliferation inhibition rates. The results showed that significant positive correlation between the concentration of combination treatment and cell inhibition rate was observed in CRC cells (p<0.001). The toxic effects of Corligin on the NCM460 cells were significantly reduced compared to CRC cell lines. **(D) Synergistic effects on SW480 cells exist with the combination of these two drugs.** SW480 cells were treated with a fixed ratio concentration (1:3) of Corilagin and 5-FU in combination for 48h and cell survival was assayed. **(E) Dose Effect Curve of SW480 cells treated with Corilagin and/or 5-FU for 48 hours.** The Combination Index (CI) values and drug effects (inhibition rate, Fa) were analyzed based on the Chou-Talalay method and Compusyn software. **(F) Combination Index Plot of Corilagin and 5-FU combined therapy. (G) Isobologram for Combo of Corilagin and 5-FU combined therapy.** These results showed that Corilagin and 5-FU produced a synergistic effect against SW480 cells (0.3<CI<0.7 for synergistic effect and CI<0.3 for strong synergistic effect).

**B**

**A**

**C**

#
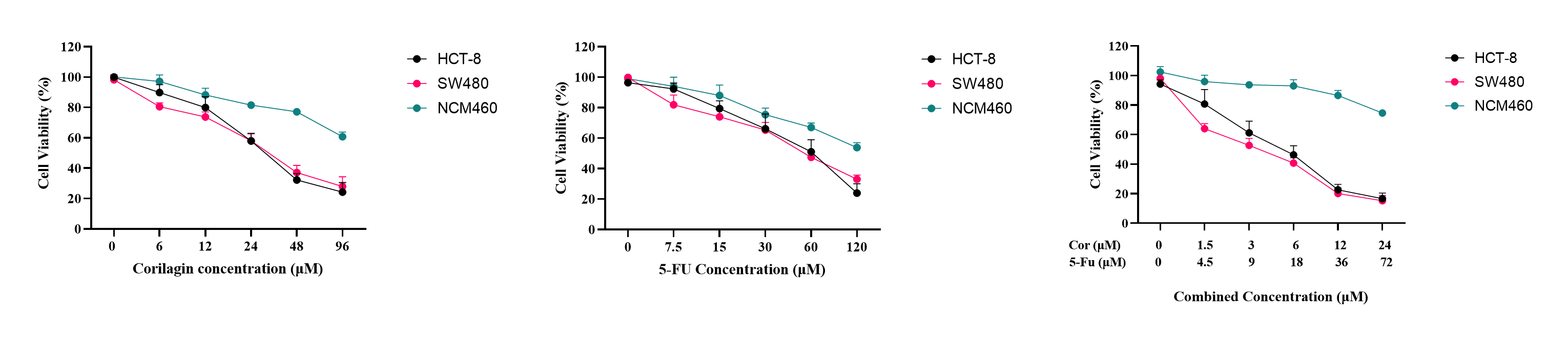

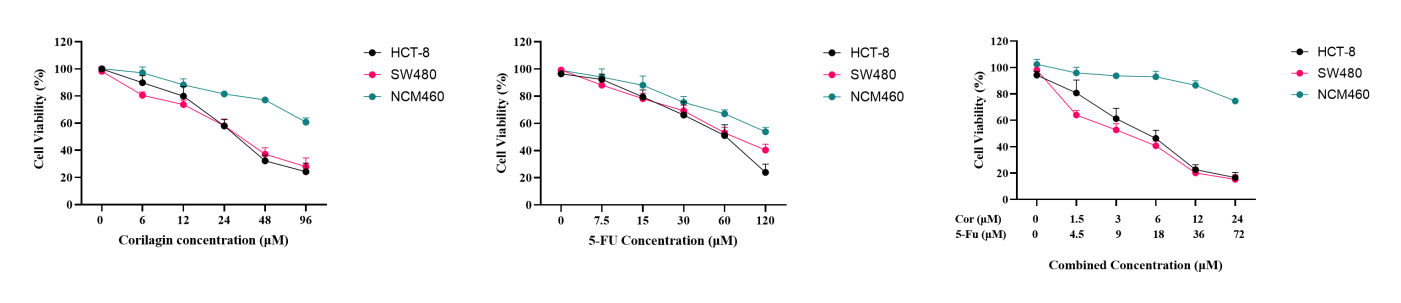

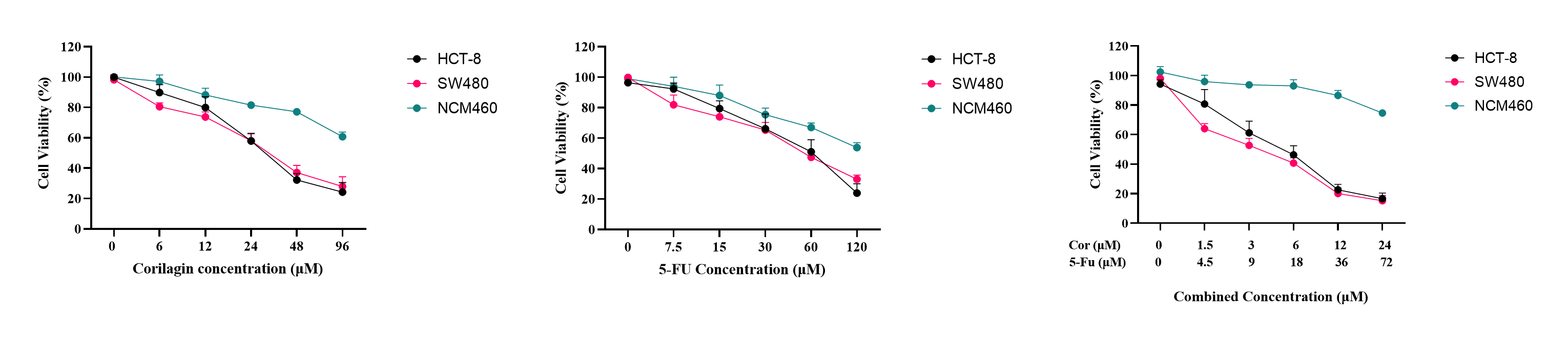

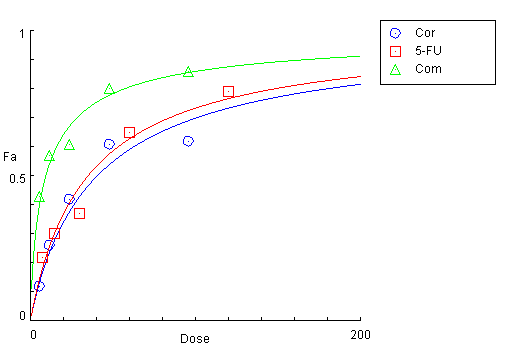

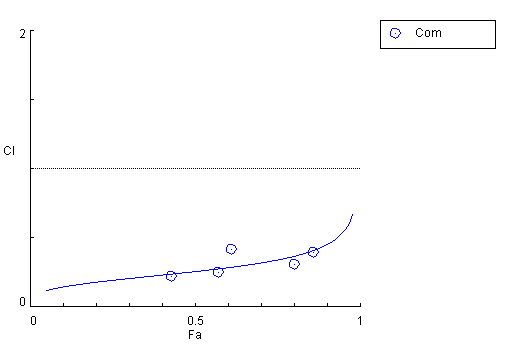

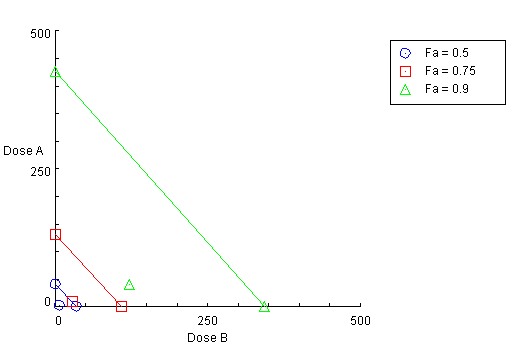


**E**

**D**

**F**

**Supplementary Fig. 4. Original Blots of Western Blotting Results for detecting CDK2 and p21 levels.**

Representative images of WB reactions with protein extract from HCT-8 cells in corligin and/or 5-FU treatment, showing the modulation of the expression of p21 (CDK inhibitor 1A, 21kD) **(A)** and CDK2 (cyclin-dependent kinase 2, 34kD) **(B)** involved in the G1-S transition of the cell cycle. β-actin protein (37kD) was used as the loading control. The results showed that the combination treatment resulted in a significant down-regulation of CDK2 protein levels and a significant up-regulation of p21 protein levels.

**A**

Control

Corilagin

Cor+5-FU

5-FU


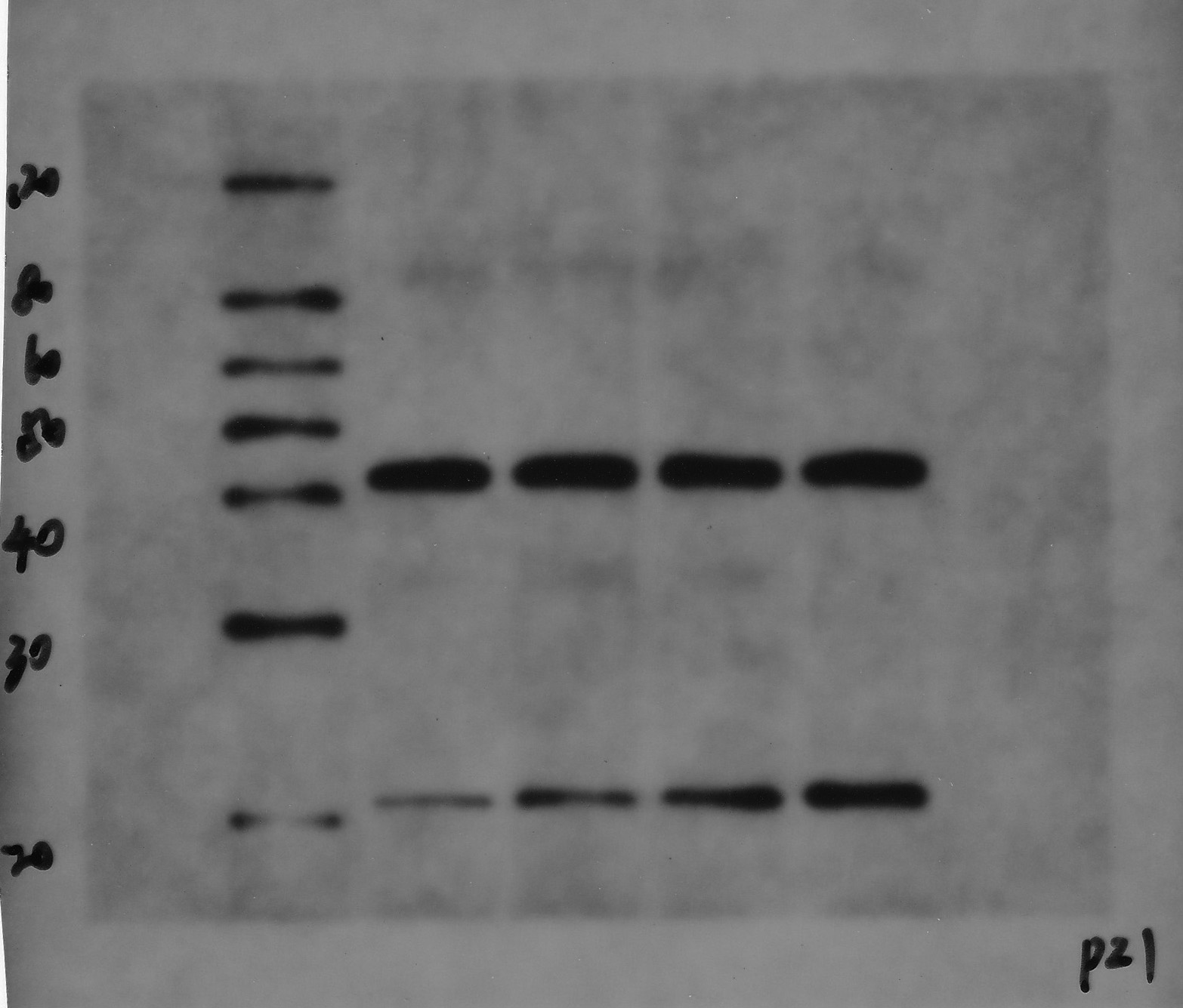


β-actin, 42kD

P21, 21kD

**B**

Control

Corilagin

Cor+5-FU

5-FU

**
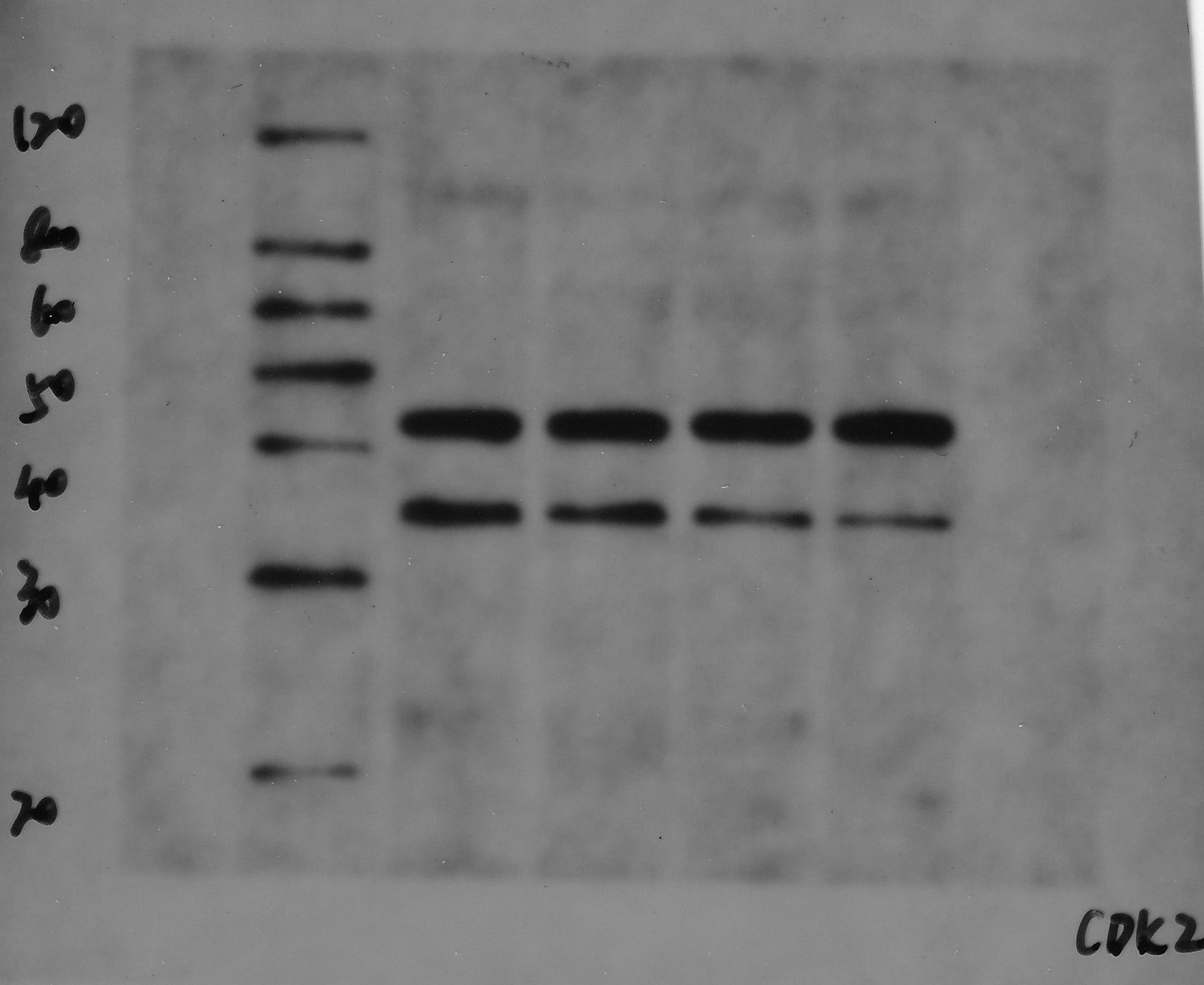
**

β-actin, 42kD

CDK2, 34kD

**Supplementary Fig. 5. Original Blots of Western Blotting Results for detecting GRP78 levels.**

Representative images of WB reactions with protein extract from HCT-8 cells in corligin and/or 5-FU treatment, showing the modulation of the expression of GRP78 protein (glucose-regulated protein, 78kD), and GAPDH protein (glyceraldehyde-3-phosphate dehydrogenase, 37kD) was used as the loading control.

Control

Corilagin

Cor+5-FU

5-FU


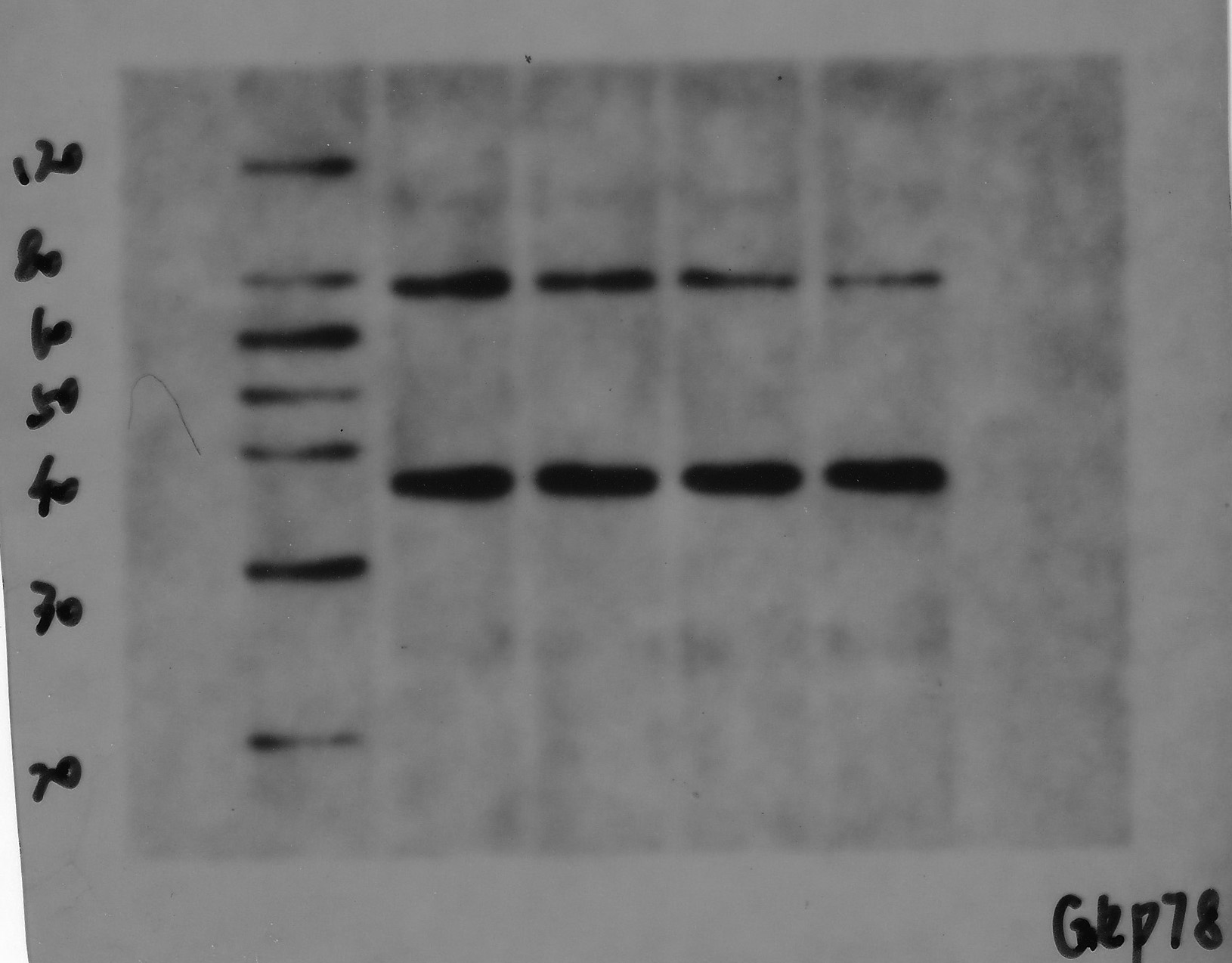


GAPDH, 37kD

GRP78, 78kD
